# Popcorn is more satiating than potato chips in normal-weight adults

**DOI:** 10.1186/1475-2891-11-71

**Published:** 2012-09-14

**Authors:** Von Nguyen, Lisa Cooper, Joshua Lowndes, Kathleen Melanson, Theodore J Angelopoulos, James M Rippe, Kristin Reimers

**Affiliations:** 1 Rippe Lifestyle Institute, 215 Celebration Place, Celebration, , FL, 34747, USA; 2 Department of Nutrition and Food Sciences, University of Rhode Island, Knigston, RI, 02881, USA; 3 Center for Lifestyle Medicine and Department of Health Professions, University of Central Florida, Orlando, FL, 32816, USA; 4 Rippe Lifestyle Institute, 21 N. Quinsigamond Avenue, Shrewsbury, MA, 01545, USA; 5 ConAgra Foods, 5 ConAgra Drive, Omaha, NE 68102, USA

**Keywords:** Popcorn, Satiety, Hunger, Fullness, Snack, Energy intake, Energy compensation, Weight management

## Abstract

**Background:**

Strategies that may increase compliance to reduced energy intakes are needed to reduce the health burden of obesity. Conflicting evidence exists regarding the effects of snacking on satiety and energy intake.

**Methods:**

This study compared short-term satiety from two common snack foods, low fat popcorn or potato chips. Using a counterbalanced within-subject design, 35 normal weight non-smoking participants (17 men, 18 women) ages 20–50 years (mean age 33 ± 11, BMI 23 ± 2 kg/m^2^) consumed four conditions each: 200 mL of water (control), one cup (4 g, 15 kcal) popcorn, 6 cups (27 g, 100 kcal) popcorn, and one cup (28 g, 150 kcal) potato chips, each with 200 mL water. Participants rated their hunger, satisfaction, prospective consumption, and thirst on 100 mm visual analogue scales 30 minutes after commencement of snack consumption. In addition, post-snack energy intake from an ad libitum meal (amount served less amount remaining) was measured, and the test food and meal combined energy intake and energy compensation were calculated.

**Results:**

Participants expressed less hunger, more satisfaction, and lower estimates of prospective food consumption after six cups of popcorn compared to all other treatments (P < 0.05). Energy compensation was 220% ± 967%, 76% ± 143% and 42% ± 75% after one cup popcorn, six cups popcorn and one cup potato chips, respectively. Combined energy intake was significantly greater (P < 0.01) during the potato chips condition (803 ± 277 kcal) compared to control (716 ± 279 kcal) or popcorn conditions (698 ± 286 kcal for one cup and 739 ± 294 kcal for six cups). Combined energy intakes from both popcorn conditions were not significantly different than control (p > 0.05).

**Conclusion:**

Popcorn exerted a stronger effect on short-term satiety than did potato chips as measured by subjective ratings and energy intake at a subsequent meal. This, combined with its relatively low calorie load, suggests that whole grain popcorn is a prudent choice for those wanting to reduce feelings of hunger while managing energy intake and ultimately, body weight.

## Introduction

To help quell the burgeoning obesity epidemic, researchers have devoted considerable effort to understand functional attributes of foods and nutrients that may influence energy intake. Satiety, the sensations that determine the intermeal period of fasting [1], is affected by many variables including food volume, weight, energy, macronutrient content, physical form, type, and variety [1-8]. The impact of satiety on long term energy balance and ultimately weight management is debated [9], but satiety is nonetheless a measurable construct that reliably influences ingestive behaviors, thus creating a connection to energy balance and ultimately body weight [10-13].

Understanding the relationship of snack foods to satiety and energy balance is important because snack foods now contribute approximately one fourth of U.S. adults’ total daily energy intake, similar to that of lunch and greater than the energy contribution of breakfast [14]. Snack foods are typically described as being more energy dense and less nutrient dense than foods consumed at meals [15,16], but this is not necessarily the case for snack foods such as vegetables, fruits or whole grain foods such as popcorn. The heterogeneity of snack foods, and the potentially diverse impact of snacks on eating behaviors and on the overall diet pattern has not received a great deal of attention. Because snacking has become an increasingly important segment of the American eating pattern, understanding the attributes of various snacks has become more relevant. The purpose of the current research was to compare the effects on satiety of two snacks, popcorn and potato chips. Potato chips are the most commonly consumed savory snack food in America [17], eaten approximately 50 times per person annually [18]. Popcorn is the 10th ranked savory snack in the U.S., eaten approximately nine times per person annually [18], and has been shown to have a beneficial association with whole grain and fiber intake among those who consume it [19].

## Methods

### Participants

Thirty five healthy non-smoking females (n = 18) and males (n = 17) ages 20–50 years (mean age 33 ± 11) with BMI 18–25 kg/m^2^ (mean BMI 23 ± 2) were recruited primarily by newspaper advertisement to voluntarily participate in the study that will provide data on the effects on satiety (appetite) after consuming popcorn and potato chips in varying amounts. Interested individuals were screened via a phone interview for exclusion criterion that included allergy to or disliking of the test foods, smoking, prescription medications, special diet, diabetes, gastrointestinal disorders, eating disorders, and pregnancy, lactation, or trying to become pregnant. Prospective participants were also screened for dietary restraint (score > 10 was disqualifying), disinhibition and perceived hunger during their initial visit by administration of the Eating Inventory [20]. Using standard procedures, body mass and height were measured (Scale Tronix, Wheaton, IL), then BMI calculated to confirm prospective participants met the BMI criteria. The Florida Hospital Institutional Review Board, Celebration Florida, approved the study and all participants provided written informed consent at the research center before beginning the study.

### Design/Protocol

Using a within-subject counterbalanced design, participants consumed each of four test foods 30 minutes prior to an ad libitum meal. Test days were scheduled at least three days apart and all participants completed four treatments within 60 days. Participants were instructed to refrain from alcohol consumption and exercise the day prior to each test day and to fast for 12 hours overnight. Upon arriving at the research facility at 9:00 a.m., participants were seated in secluded rooms where they stayed until approximately 1:00 p.m. Shortly after arrival, the participants consumed a 500 kcal (men) or 400 kcal (women) standard meal within 15 minutes, consisting of 44 g ready-to-eat cereal, 462 g whole milk, and 130 g orange juice for men and 33 g ready-to-eat cereal, 371 g whole milk and 130 g orange juice for women. Caloric distribution for the meal was 55% carbohydrate, 15% protein and 30% fat. One cup (240 mL) of coffee or tea was provided for those who routinely consumed these beverages in the morning. Participants were instructed to consume the entire meal, and all were compliant in doing so.

On the first visit, participants also received a 500 mL bottle of water, which they were allowed to consume ad libitum between the morning meal and one hour prior to consuming the test food. The amount consumed was provided at all subsequent visits. Prior to receiving the test food, participants were allowed to sit quietly and to read, but were not allowed access to any materials that contained reference to food, eating, dieting, or body weight. Thirty minutes prior to the ad libitum meal, participants were served the relevant test food and were required to consume it within 15 minutes. The ad libitum meal was served at 12:30 p.m. and participants were invited to eat as much as they liked with no time restriction.

### Test foods and Ad libitum meal

The control condition was 200 mL of water. The test conditions consisted of one cup (4 g, 15 kcal) or six cups (27 g, 100 kcal) of 94% fat free microwave popcorn, or one cup (28 g, 150 kcal) of potato chips, all served with 200 mL water. These quantities were chosen to compare both equal volumes (one cup) of popcorn and potato chips as well as labeled serving sizes. The labeled individual serving sizes on these packaged products were 27 g for a 100 kcal mini bag of popcorn (approximately six cups) and 28 g for one serving (approximately one cup) of potato chips. The ad libitum meal consisted of a generous pre-weighed portion of commercially prepared macaroni and cheese and water (500 mL). Participants were invited to eat as much as they wanted and were invited to request more if they so desired. The macronutrient energy distribution and fiber content of the test foods and ad libitum meal are shown in Table 1.

**Table 1:** Nutrient composition of test foods and ad libitum meal as served

	**200 mL water**	**1 cup popcorn + 200 mL water**	**6 cups popcorn + 200 mL water**	**1 cup potato chips + 200 mL water**	**Macaroni and cheese entrée**
Food Weight, g	--	4	27	28	645
Kcal	0	15	100	150	1000
Fat, g	0	<1	1.5	9.5	49
Fat, % kcal	0	14	14	57	44
Carbohydrate, g	0	3	21	15	97
Carbohydrate, % kcal	0	74	74	40	39
Fiber, g	0	0.8	3	1	6
Protein, g	0	<1	3	1	43
Protein, % kcal	0	12	12	3	17

### Measures

Satiety was measured using visual analogue scales (VAS), as well as measures of subsequent energy intake and energy compensation [21]. Participants rated hunger, satisfaction, how much food they thought they could eat (prospective consumption) and thirst on 100 mm VAS anchored with extreme ratings of “not at all” vs. “extremely” to describe how hungry, satisfied or thirsty they were and “nothing” or “vast quantities” to describe how much they could eat. Palatability was assessed using VAS with ratings for extremes of pleasant, salty, texture, bitter and taste. Participants received instruction for and practiced completing the VAS prior to the study. At each of the four trials, palatability was rated after the first bite of the test food and satiety rated 30 minutes later, immediately before access to the ad libitum meal. Intake of the ad libitum meal was calculated by weighing to the nearest 1.0 gram the starting meal weight and subtracting the remaining food weight (Tanita scale model 1458N, Arlington Heights, IL). Energy (kcal/g) of the macaroni and cheese was based on labeled kcal content. Combined energy intake was determined by summing the energy intake of the test food plus the ad libitum meal. Percent energy compensation of the test foods at the subsequent ad libitum meal was calculated as: (meal kcal intake during control condition – meal kcal intake during test condition) / test food kcal content x 100.

### Statistical analysis

One way ANOVA with repeated measures was used to test differences across the treatments for each dependent variable. Pairwise comparisons were made when necessary via dependent t-tests, applying the Bonferroni adjustment. Statistical significance was set at p <0.05.

## Results

Compared to control, all three test conditions resulted in significantly less hunger, more satisfaction, lower estimates of prospective food consumption, and more thirst (p < 0.01, Table 2). Six cups of popcorn produced greater satiety ratings compared to both one cup of popcorn and one cup of potato chips (p < 0.05).

**Table 2:** VAS palatability and satiety ratings for control, popcorn and potato chip conditions

	**Water control**	**Popcorn**	**Popcorn**	**Potato chips**
		**One cup**	**Six cups**	**One cup**
**Rating, mm mean ± standard deviation**				
How pleasant is this food?	21.7 ± 25.9	51.3 ± 27.1***	57.7 ± 23.2***	51.4 ± 21.5***
How salty is this food?	4.2 ± 10.7	49.2 ± 23.0***†††	57.9 ± 26.8***	67.2 ± 26.3***
How bitter is this food?	2.6 ± 3.4	11.6 ± 14.4**	10.8 ± 15.7**	8.9 ± 13.2**
How much do you like the texture of this food?	31.3 ± 26.7	59.4 ± 25.6***	58.2 ± 21.5***	56.7 ± 17.9***
How much do you like the taste of this food?	17.3 ± 23.7	56.8 ± 25.7***	59.4 ± 22.9***	57.6 ± 22.3***
How hungry are you?	73.5 ± 18.7	63.9 ± 17.4***†††	47.7 ± 20.5***	60.1 ± 21.9***††
How satisfied are you?	22.4 ± 17.0	36.1 ± 19.3***††	47.9 ± 20.5***	39.6 ± 21.9***†
How much could you eat?	71.7 ± 16.1	63.3 ± 18.0***††	49.3 ± 19.5***	57.9 ± 20.1***†
How thirsty are you?	27.6 ± 23.8	36.4 ± 22.6**†	43.7 ± 25.6**	44.5 ± 25.6***

** Significantly different from water (P < 0.01).

*** Significantly different from water (P < 0.001).

† Significantly different from six cups popcorn (P < 0.05).

†† Significantly different from six cups popcorn (P < 0.01).

††† Significantly different from six cups of popcorn (P < 0.001).

The visual analog scales were 100mm in length. How pleasant, salty and bitter scales were anchored by “not at all” and “extremely” and the texture and taste scales were anchored by “dislike extremely” and “like extremely.” The satiety VAS was measured 30 minutes after commencement of test food. How hungry, satisfied, and thirsty scales were anchored by “not and all” and “extremely.” How much could you eat scale was anchored by “nothing” and “vast quantities”.

Ad libitum meal consumption was significantly decreased after consumption of six cups of popcorn and one cup of potato chips compared to control (Table 3). The combined energy intake (test food plus meal) of each popcorn condition was not different from control and significantly less than (p < 0.01) the combined energy intake of the potato chips condition. The extent of energy compensation from potato chips was 42% ± 75% (63 ± 112 kcal) compared to 76% ± 143% (76 ± 143 kcal) for six cups popcorn and 220% ± 967% (33 ± 145 kcal) for one cup popcorn.

**Table 3:** Combined energy intakes during control, popcorn and potato chip conditions

	**Water control**	**Popcorn**	**Popcorn**	**Potato chips**
		**One cup**	**Six cups**	**One cup**
**mean ± standard deviation**				
Water, ml	338 ± 194	351 ± 256	333 ± 202	321 ± 196
Macaroni and cheese lunch, kcal	716 ± 279	683 ± 286	639 ± 294††	653 ± 277††
Test food, kcal	0	15	100	150
Combined energy intake, kcal	716 ± 279***	698 ± 286***	739 ± 294**	803 ± 277
Energy Compensation, kcal		33 ± 145	76 ± 143	63 ± 112
Energy Compensation, %		220 ± 967	76 ± 143	42 ± 75

** Significantly different from one cup potato chips (P < 0.01).

*** Significantly different from one cup potato chips (P < 0.001).

†† Significantly different from control (P < 0.01).

## Discussion

These data indicate that popcorn resulted in greater satiety than potato chips. The six cup popcorn condition exhibited greater satiety than potato chips based on the outcome measures of VAS ratings and combined energy intake. One cup popcorn (15 kcal) did not elicit stronger VAS responses than one cup potato chips (150 kcal), but did elicit similar VAS responses (not statistically different) despite the 10-fold energy difference, suggesting that 15 kcal of popcorn was as satiating as 150 kcal of potato chips. Additionally, the one cup popcorn condition resulted in less combined energy intake and incomplete mean energy compensation. These data in their totality indicate that both popcorn conditions resulted in greater satiety than potato chips.

Several attributes of popcorn may contribute to its satiating effect at a relatively low energy level, for example, its low energy density [3]. The energy density of the 94% fat free popcorn (3.7 kcal/g) is 31% lower than that of potato chips (5.4 kcal/g). Volume is likely another satiety-promoting quality of popcorn. Starch expansion during the popping process produces a foam-like matrix with a large surface-area to mass ratio. This trait, combined with popcorn’s irregular shape, leads to a food with a high volume per unit weight. High volume, due to incorporating air into food and due to irregular shape, has been shown to increase satiety [2,4,22]. Additionally, the proportionality of macronutrients may contribute to satiety, as prior research has shown that fat is less satiating than carbohydrate or protein [23-25]. The higher fat content of potato chips may have contributed to lower VAS scores and less compensation. Finally, the participants were not blinded to the volume or appearance of the snacks, so the visual differences among the snacks may have influenced perceptions of hunger, satisfaction and prospective consumption. Any combination of these characteristics of popcorn could potentially contribute to its effect on short-term satiety despite the relatively low calorie provision.

Snacks that offer relatively higher levels of satiety may be beneficial for weight management provided the snack does not contribute to greater overall energy intake. One of the primary issues that has been identified in relationship to snacking, satiety and energy intake is the inability to fully compensate for the energy consumed as snacks [15,26,27]. Evidence shows that snacks consumed in a non-hungry state do not impact satiety or reduce energy intake at the subsequent meals [15,26-30] and cross-sectional and longitudinal studies suggest that snacking is associated with weight gain and or obesity [16,31,32]. Energy dense, highly palatable foods such as cookies, cakes, desserts, and candies are associated with higher energy intakes in obese adults [16]. In contrast, compensation for popcorn was observed in the current study, resulting in overall energy intake not different from having no snack. This finding is supported by previous population-based research that shows popcorn consumption is not related to increased body mass index [19]. In addition, longitudinal [6] and weight loss interventions [33-36] indicate that snacking has a neutral or positive effect on energy intake or body mass index. For example, provision of up to three snacks daily as part a weight loss diet had a neutral effect on weight change [33-35].

The inconsistency in results about snacking may be attributed to many variables such as inconsistent definition of a snack, whether the snack is consumed during a non-hungry state, the timing of the snack around the meal, whether the snacking is part of usual intake or a reduced calorie diet, the population studied, and the attributes of the food itself (energy density, volume, macro- and micronutrient composition and physical form). The types of foods consumed as snacks are extremely broad, ranging from nutrient dense fruits, vegetables and whole grains, to energy dense desserts and confections. As such, while some snacks do not show nutrient benefit, some snacks have been shown to improve diet quality due to higher fruit, vegetable and fiber intakes [37,38]. Population data show that individuals who consume popcorn, compared to those who do not, have significantly greater intakes of whole grain, fiber, and magnesium [19]. To strengthen future research, the heterogeneity of snacks and snacking behaviors should be considered and controlled to better characterize the role of snacking as it relates to energy balance and weight management.

### Limitations and future research

This study has several limitations and stimulates additional research questions. The generalizability of this study is limited due to the sample including only normal weight men and women less than 50 years of age. The findings may vary on overweight, obese or older adults and children. Serving the meal 30 minutes after commencement of the test food was designed to capture the satiety effects of the relatively low energy loads of the test foods (15 kcal to 150 kcal), but this relatively short time interval may not represent a typical snack-meal pattern. Thus, the results of this study support the short-term satiety attribute of popcorn relative to potato chips, but cannot be generalized to satiety, energy compensation, or energy reduction over longer periods of time.

Future research should consider testing a wide variety of snack foods, including higher fat popcorn. It would also be of interest to compare eucaloric portions of popcorn with various other snacks. One question spurred by this current study is whether a 100 kcal portion of other commonly consumed snacks would lead to more complete energy compensation, as was observed with popcorn. Similarly, the effects of popcorn at energy loads greater than 100 kcal are unknown. Timing of the test food before the meal is another area of interest. A study that models a typical snacking pattern would be useful to build upon the current findings. Finally, future research should be carried out for longer periods of time to ascertain whether energy compensation is maintained throughout the course of the day.

## Conclusion

Foods or nutrients that enhance satiety are considered one of many factors that affect energy intake regulation [10-12]. A snack that reduces the burden of hunger, while not increasing overall energy intake, may offer a benefit for individuals trying to maintain or achieve energy balance. In this study, low fat popcorn was shown to exert greater short-term satiety than potato chips. The satiety attribute, combined with popcorn’s other favorable characteristics of being a whole grain, high fiber, nutrient dense snack, support popcorn as beneficial snack choice in the context of healthy weight management.

## Competing interests

This research was supported by ConAgra Foods, Inc., makers of Orville Redenbacher and Act II popcorn. K. Reimers is an employee of ConAgra Foods.

## Authors contributions

VN participated in the design of the study, carried out the dietary analysis and helped draft the manuscript. LC participated in the design of the study and carried out the dietary analysis. JL participated in the design of the study, supervised data entry, performed the statistical analyses, and helped draft the manuscript. KM participated in the design of the study and helped draft the manuscript. TA participated in the design of the study, had the responsibilty of the statistical analyses, and helped draft the manuscript. JR participated in the design of the study and helped draft the manuscript. KR participated in the design of the study and helped draft the manuscript. All authors read and approved the final manuscript.
